# Improvement of carrier diffusion length in silicon nanowire arrays using atomic layer deposition

**DOI:** 10.1186/1556-276X-8-361

**Published:** 2013-08-23

**Authors:** Shinya Kato, Yasuyoshi Kurokawa, Shinsuke Miyajima, Yuya Watanabe, Akira Yamada, Yoshimi Ohta, Yusuke Niwa, Masaki Hirota

**Affiliations:** 1 Department of Physical Electronics, Tokyo Institute of Technology, Meguro-ku, Tokyo 152-8552, Japan; 2 PRESTO, Japan Science and Technology Agency (JST), Tokyo 102-0076, Japan; 3 Photovoltaics Research Center (PVREC), Tokyo Institute of Technology, Tokyo 152-8550, Japan; 4 Advanced Materials Laboratory, Nissan Research Center, Yokosuka, Kanagawa 237-8523, Japan

**Keywords:** Silicon nanowire, Passivation, Atomic layer deposition, Lifetime, Simulation, Diffusion length, 62.23.Hj, 77.55.df, 81.65.Rv

## Abstract

To achieve a high-efficiency silicon nanowire (SiNW) solar cell, surface passivation technique is very important because a SiNW array has a large surface area. We successfully prepared by atomic layer deposition (ALD) high-quality aluminum oxide (Al_2_O_3_) film for passivation on the whole surface of the SiNW arrays. The minority carrier lifetime of the Al_2_O_3_-depositedSiNW arrays with bulk silicon substrate was improved to 27 μs at the optimum annealing condition. To remove the effect of bulk silicon, the effective diffusion length of minority carriers in the SiNW array was estimated by simple equations and a device simulator. As a result, it was revealed that the effective diffusion length in the SiNW arrays increased from 3.25 to 13.5 μm by depositing Al_2_O_3_ and post-annealing at 400°C. This improvement of the diffusion length is very important for application to solar cells, and Al_2_O_3_ deposited by ALD is a promising passivation material for a structure with high aspect ratio such as SiNW arrays.

## Background

**Figure 1:**
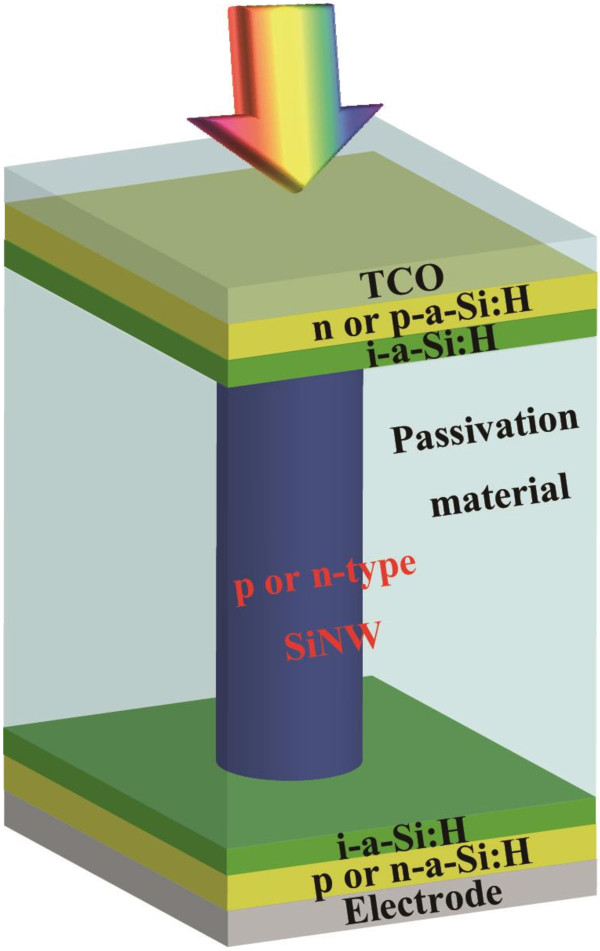
The SiNW solar cell structure that we have proposed.

Silicon nanowire (SiNW) enables us to tune the bandgap by the quantum size effect [1] and effective photo-absorption owing to strong optical confinement effect [2-4]. It is possible to apply SiNW to all-silicon tandem solar cells to utilize the broadband solar spectrum at low cost. When a crystalline silicon (1.12 eV) bottom cell is combined with a top cell with SiNW (1.74 eV) [1], all-silicon tandem solar cells have the possibility to overcome the Shockley-Queisser limit [5]. Moreover, it is expected that SiNW solar cells have higher photocurrent than crystalline silicon solar cell with the same thickness as the SiNW length owing to the higher absorption coefficient derived from optical confinement [6]. SiNW has been prepared by several top-down or bottom-up methods [7-13]. Over the past few years, many researchers have applied SiNWs to solar cells [14-19] for the purpose of optical confinement. We have proposed a SiNW solar cell with a heterojunction structure as shown in Figure 1[1]. When SiNW arrays are applied to such a solar cell, it is significantly important to reduce the surface recombination velocity of the SiNW surface by using passivation films since the large surface area of SiNWs enhances minority carrier recombination at the surface. However, there are a few reports about the passivation of silicon nanowires to reduce surface recombination velocities, which determine the performance of solar cells. Dan et al. have reported the passivation effect of a thin layer of amorphous silicon on a single-crystalline silicon nanowire prepared by the Au-catalyzed vapor–liquid-solid (VLS) process [20]. They showed that the surface recombination velocity was reduced by amorphous silicon by nearly 2 orders of magnitude. Demichel et al. have demonstrated that surface recombination velocities as low as 20 cm/s were measured for SiNWs prepared by the same process and efficiently passivated by a thermal oxidation [21]. Although these results are based on SiNWs prepared by the VLS process, considering application to solar cells, metal-assisted chemical etching is more promising [11,18,22-25] since vertical SiNW arrays can be prepared in a large area under no vacuum. However, there is no report on the deposition of passivation films and their passivation effect on SiNW arrays prepared by the MAE process. Moreover, no result has ever been reported on minority carrier lifetime in vertical SiNW arrays to estimate passivation effect. Minority carrier lifetime is the dominant factor affecting the characteristics of solar cells. Therefore, it is important to measure minority carrier lifetime to analyze the characteristics of solar cells. In our previous work, we successfully fabricated 30-nm-diameter SiNW arrays by metal-assisted chemical etching using silica nanoparticles (MACES) [23]. It is well known that aluminum oxide (Al_2_O_3_) deposited by atomic layer deposition (ALD) [26-29] and hydrogenated amorphous silicon (a-Si:H) deposited by plasma-enhanced chemical vapor deposition (PECVD) [29,30] show an excellent surface passivation effect on crystalline silicon. In this study, we investigated the deposition of a-Si:H by PECVD and Al_2_O_3_by ALD around SiNW arrays and measured the minority carrier lifetime in SiNW arrays by the microwave photo-conductivity decay (μ-PCD) method. However, the measured minority carrier lifetime was influenced by the supporting crystalline silicon substrate underneath the SiNWs. We carried out numerical simulations using PC1D (University of NSW) [31-33] simulation software to extract the minority carrier lifetime in the SiNW array layer, assuming that the SiNW layer is a homogeneous single-phase material with a minority carrier lifetime. Based on the simulation results, we proposed a simple equation to extract the minority carrier lifetime in the SiNW layer from measured minority lifetime.

## Methods

Si wafers (p-type, (100), 2 to 10 Ω cm) were used for the fabrication of SiNW arrays. The surfaces of the Si wafers were hydrophilic by modifying with an amino group. The hydrophilic Si wafers were immersed in a solution in which 30-nmsilica nanoparticles modified with a carboxyl group were dispersed at 2°C for 1 h. This process formed a dispersed silica nanoparticle layer on the Si wafer. Subsequently, a 20-nm-thick silver film was deposited on the wafers with silica nanoparticles using a DC sputtering system. After removing the silica nanoparticles by ultrasonication in deionized water, Si wafers with a nano-patterned silver film were obtained. The wafer was chemically etched using 4.8 M HF and 0.15M H_2_O_2_ at room temperature to form SiNW arrays. The remaining silver film on the bottom of the SiNW arrays was removed by HNO_3_ wet etching. Finally, the oxide layer existing on the surface of the SiNW array was removed with a HF solution. Details of the SiNW array fabrication process are shown elsewhere [23]. After the fabrication of SiNW arrays, intrinsic amorphous silicon was deposited by PECVD under the same condition as the heterojunction crystalline silicon solar cell in which the fabrication temperature is 210°C and the operating pressure is 0.3 Torr. After the deposition, the SiNW array was annealed in a forming gas at 200°C, which is the best annealing temperature for the surface passivation of our a-Si:H. On the other hand, Al_2_O_3_ was also deposited using Al(CH_3_)_3_ and H_2_O alternately at 200°C by an ALD system. After the deposition, the SiNW arrays were annealed in a forming gas at 400°C. These nanostructures of the prepared SiNW arrays were characterized by field emission scanning electron microscopy (SEM) and energy-dispersive X-ray spectroscopy (EDS) with JEOL JSM-7001F (JEOL, Tokyo, Japan). The structure of the interface between SiNW and Al_2_O_3_ was observed by transmission electron microscopy (TEM) with HITACHI H-9000NAR (HITACHI, Tokyo, Japan) and high-angle annular dark field scanning transmission electron microscopy (HAADF-STEM) with HITACHI HD-2700. Minority carrier lifetime was measured by the μ-PCD method with KOBELCO LTE-1510EP (KOBELCO, Tokyo Japan).

**Figure 2:**
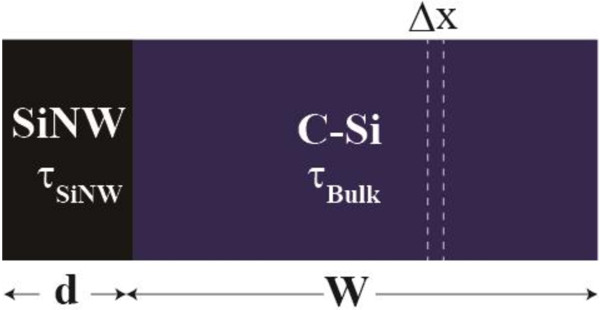
The schematic diagram of the calculation model.

To investigate the carrier lifetime in a SiNW region (*τ*_SiNW_), one-dimensional numerical simulations were carried out using PC1D. The electrical transport was calculated by solving Poisson equations and carrier continuity equations. In the simulations, we employed a simple structure in which a homogeneous single-phase material with a small carrier lifetime is stacked on a crystalline silicon substrate with a large carrier lifetime as shown in Figure 2. The homogeneous single-phase material is equivalent to the SiNW region. We calculated the effective minority carrier lifetime in the structure (*τ*_whole_) as a function of the minority carrier lifetime in the equivalent SiNW region (*τ*_SiNW_) to investigate the relationship between *τ*_whole_ and *τ*_SiNW_. *τ*_whole_ corresponds to the measured effective lifetime (*τ*_eff_). Electrical parameters used in our simulations are summarized in Table 1. The absorption coefficient of a SiNW was used with the same value as crystalline silicon because it has not been confirmed by an experiment yet. The wavelength of an incident light was 904 nm, which is the same as the wavelength of the laser used in μ-PCD measurement. Moreover, Shockley-Read-Hall recombination, Auger recombination, and band-to-band recombination were taken into account, and the surface recombination was neglected for simplification.

**Table 1:** Physical parameters for lifetime estimation based on our simple calculation model and PC1D

**Symbol**	**Parameter**	**Silicon nanowire**	**Bulk silicon**
*d*, *W*	Length, thickness	10 μm	190 μm
*Ε*	Dielectric constant	11.4	11.4
Eg	Energy gap (eV)	1.12	1.12
χ	Electron affinity (eV)	4.05	4.05
Dt	Trap level	0	0
*τ*_e0_, *τ*_h0_	Carrier lifetime	0.05 to 1.5 μs	1 ms
*μ*_e_	Electron mobility (cm^2^/(Vs))	1,104	1,104
*μ*_h_	Hole mobility (cm^2^/(Vs))	424.6	424.6
*N*_A_	Accepter concentration (cm^−3^)	1 × 10^16^	1 × 10^16^

## Results and discussion

**Figure 3:**
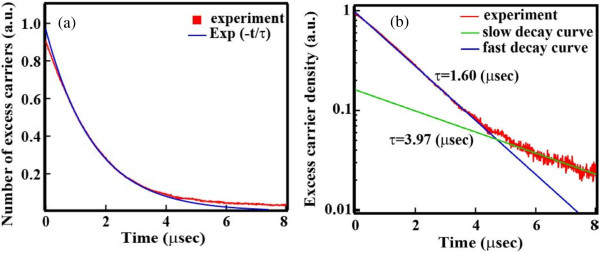
**Transient response of excess carrier density in a SiNW array on bulk silicon. (a)** Linear scale. **(b)** Logarithmic scale.

**Figure 4:**
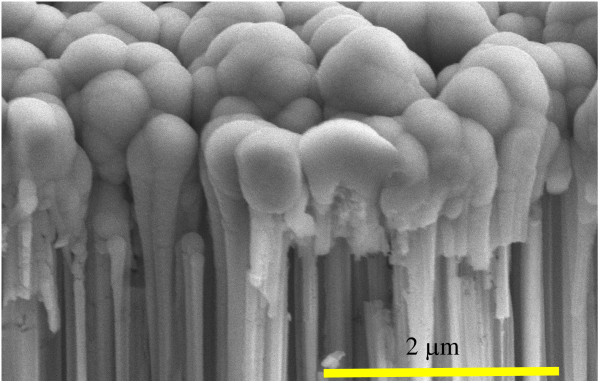
Cross-sectional SEM image of an a-Si:H thin film deposited on a SiNW array.

**Figure 5:**
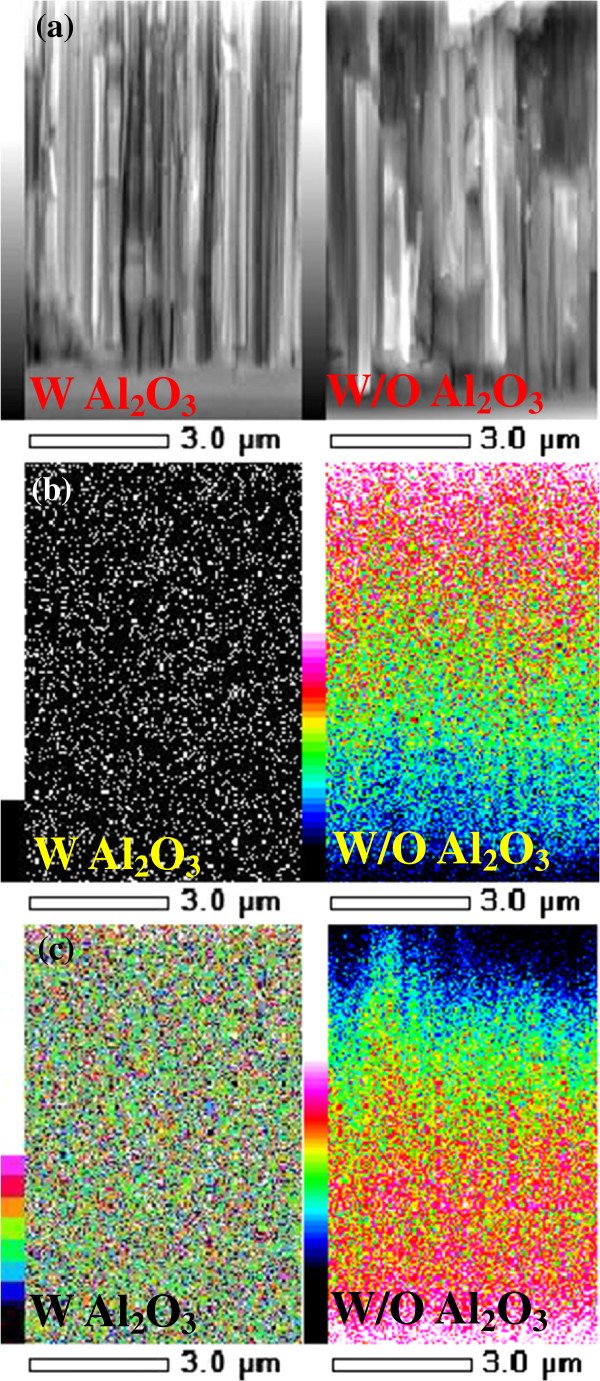
**SEM image and EDS mapping of SiNW without and with Al_2_O_3_. (a)** Cross-sectional SEM image of SiNWs without and with Al_2_O_3_. EDS mappings of **(b)** Al and **(c)** Si corresponding to the SiNWs shown in **(a)**, respectively.

**Figure 6:**
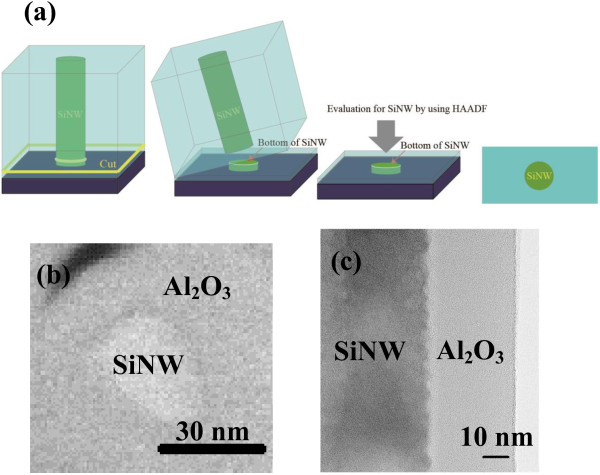
**HAADF-STEM and TEM images of the SiNW with Al_2_O_3_. (a)**The procedure on how to measure the HAADF-STEM image. **(b)** Cross-sectional HAADF-STEM image of a SiNW cut into a round slice at the bottom of the SiNW array. **(c)** Cross-sectional TEM image of the interface between the SiNW and Al_2_O_3_.

The decay curve of SiNW arrays fabricated by MACES was successfully obtained from μ-PCD measurement, as shown in Figure 3a. From Figure 3b, we confirmed that the decay curve consisted of two components, which were fast-decay and slow-decay components. At present, the origin of the second slow-decay component is not clear. A possible explanation is that the slow decay originates from minority carrier trapping effect at the defect states on the surface of the SiNW arrays. As a result of fitting to exponential attenuation function, the *τ*_eff_ of the SiNW arrays on the Si wafers is found to be 1.6 μs. This low *τ*_eff_ reflects the large surface recombination velocity at the surface of the SiNW arrays because we used high-quality crystalline silicon wafer as starting materials. To improve *τ*_eff_, passivation films were deposited on the SiNW arrays. In the case of the a-Si:H passivation film, *τ*_eff_ was not improved since only a small part of the SiNW arrays was covered with the a-Si:H film. The a-Si:H thin film was deposited only on top of the SiNW array owing to the high density of SiNWs as shown in Figure 4. This reason can be explained according to the studies of Matsuda et al., in which they reported about the deposition of a-Si:Hon trench structure by PECVD [34,35]. The concentration of precursors related with a silane gas decreased as their position on the SiNW moved farther from the plasma region, suggesting that the precursors could not reach the bottom of the SiNWs. That is why the a-Si:H thin film was deposited only on top of the SiNW array. In fact, the interspace between our fabricated SiNWs could not be embedded owing to the very narrow gap at around 20 nm. On the other hand, in the case of SiNW arrays covered with the as-deposited Al_2_O_3_ film, the *τ*_eff_ increased to 5 μs. That is because the surface of the SiNW arrays was successfully covered with Al_2_O_3_. In Figure 5a, the cross-sectional SEM images of the SiNW array before and after the deposition of an Al_2_O_3_ passivation film are shown. After the deposition of Al_2_O_3_, the dark contrast owing to the gap between SiNWs disappeared, suggesting that the Al_2_O_3_ film macroscopically covered SiNWs. Figure 5b,c shows the EDS mappings of aluminum and silicon, respectively. White and black signals show a maximum and minimum value, respectively. Note that the signal of aluminum was detected on the bottom of SiNWs after Al_2_O_3_ deposition, although the signal was not detected before the deposition. However, the Al intensity around the bottom was weaker than the one at the top. From a SEM image, the shape of SiNWs around the top is needle-like and the gap between SiNWs is about several hundred nanometers, although the gap around the bottom is about several ten nanometers (not shown). Therefore, the intensity of Al is higher around the top. These results also suggest that the Al_2_O_3_ film macroscopically covered SiNWs from the top to the bottom. To investigate the microscopic structure of the interface between a SiNW and Al_2_O_3_, TEM and HAADF-STEM observations were carried out. Figure 6a,b shows a schematic diagram on how to fabricate the sample for HAADF observation and a HAADF image of the SiNW cut into a round slice at the bottom of the SiNW, respectively. The contrast of a HAADF image is proportional to the square of the atomic number and becomes brighter with increasing atomic number. The contrast between the SiNW and Al_2_O_3_ is very clear in the figure. It should be noted that there is no gap at the interface. In Figure 6c, the uniform thickness of Al_2_O_3_ can be seen and is about 30 nm, which is enough for the passivation of crystalline silicon solar cells [29]. The uniform deposition on the SiNW arrays is due to the excellent surface coverage of ALD techniques. From these results, the Al_2_O_3_ film deposited by the ALD method was able to cover the SiNW arrays up to the bottom. Since the fine interface between a SiNW and Al_2_O_3_ was formed and dangling bonds on the surface were modified by oxygen, the minority carrier lifetime in the SiNW arrays was improved.

For further improvement of carrier lifetime, annealing of the SiNW arrays embedded in Al_2_O_3_ was carried out. It was reported that negative fixed charge density at the interface of Al_2_O_3_/p-type c-Si increased from 1.3 × 10^11^ to 2.45 × 10^12^ cm^−2^ by annealing at 400°C [36]. This increase of fixed charge density increases electric field at the Al_2_O_3_/SiNW interface. The electric field effectively repels minority carrier from the interface, resulting in the increase of minority carrier lifetime in the SiNW arrays. However, if a SiNW has perfect cylindrical symmetry, and Al_2_O_3_ with negative fixed charge is deposited on the surface uniformly, the electric field in the SiNW will be cancelled due to the symmetry of the electric field. Since in this case the effect of field effect passivation cannot be obtained, the effective lifetime will not be improved by annealing. To confirm the hypothesis, we tried to anneal the SiNW arrays with Al_2_O_3_ at 400°C. As a result, our SiNW samples also showed improvement of effective minority carrier lifetime, as well as a flat c-Si substrate passivated by Al_2_O_3_ layers, after annealing at 400°C. The *τ*_eff_ was found to be 27 μs. From this result, we conclude that since the prepared SiNWs do not have a perfect cylindrical symmetry, the effect of field effect passivation can be successfully obtained. Since negative charge density in the Al_2_O_3_ was increased by annealing at 400°C, the effective lifetime was also improved.

**Figure 7:**
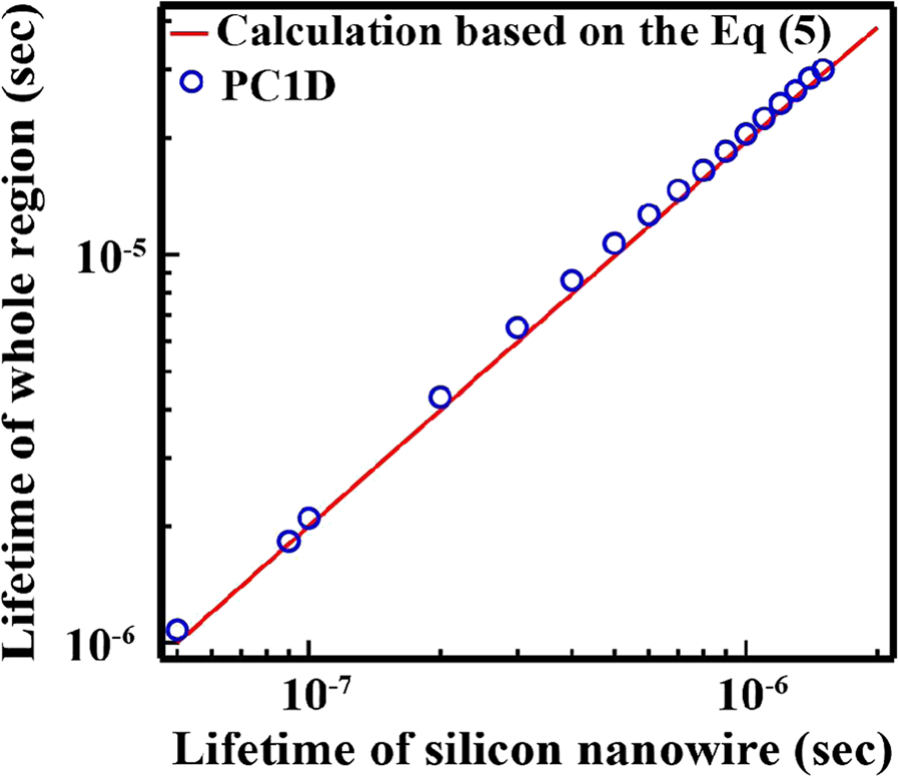
**The calculated carrier lifetime.** Carrier lifetime in only a SiNW as a function of the carrier lifetime in the whole region by calculation based on Equation 5 and PC1D.

Although *τ*_eff_ of the SiNW arrays on the Si wafers were successfully obtained, we cannot consider these lifetimes as the lifetime of the SiNW region (*τ*_SiNW_) due to the influence of the Si wafers. Therefore, we tried to extract *τ*_SiNW_ from *τ*_eff_ using PC1D simulation. PC1D simulations revealed that *τ*_eff_ was significantly influenced by the Si wafers. The calculated *τ*_whole_ which is equivalent to the measured *τ*_eff_ is 20 times higher than *τ*_SiNW_, as shown in Figure 7. These simulations clearly indicate that the measured *τ*_eff_ is completely different from *τ*_SiNW_.

We proposed a simple equation to extract *τ*_SiNW_ from *τ*_eff_ without numerical simulations. In the simulations of PC1D, minority carrier continuity equations were used. In general, the terms of drift, diffusion, recombination, and photogeneration have to be considered in the continuity equations. However, the terms of electric field and photogeneration can be eliminated. In μ-PCD measurement, a decay of excess carrier density is measured after stopping a laser irradiation. Therefore, photogeneration can be neglected. Although negative charge in Al_2_O_3_ can form electric field on the surface of SiNWs, the influence of the electric charge on excess carriers is limited only on the surface. Therefore, in this calculation, electric field was neglected for simplification. It was assumed that carriers were generated uniformly in the whole region because the carrier density remained alternated by time variation from the resulting PC1D. Therefore, the term diffusion current can be also neglected, and only the recombination current in continuity equations can be considered. The recombination current in infinitesimal difference Δ*x*(*J*) is given by

(1) $$J=q\frac{\mathrm{dn}}{\mathrm{dt}}\mathrm{\Delta x}=-q\frac{n}{\tau }\mathrm{\Delta x}$$

where *q* is the elementary charge, *n* is the density of electron, and *τ* is the lifetime. If the lifetimes of SiNW and bulk silicon are taken in account, the recombination current in the whole region is represented by

(2) $$J_{\mathrm{total}}=J_{\mathrm{SiNW}}+J_{\mathrm{Bulk}}=-\mathrm{en}\left(\frac{d}{\tau_{\mathrm{SiNW}}}+\frac{W}{\tau_{\mathrm{Bulk}}}\right)$$

where *d* is length the of a SiNW, *W* is the thickness of bulk silicon, *τ*_SiNW_ is the lifetime of a SiNW, and *τ*_Bulk_ is the lifetime of bulk silicon. On the other hand, when the effective lifetime is considered as the whole region lifetime (*τ*_whole_), the recombination current in the whole region is given by

(3) $$J_{\mathrm{total}}=-\mathrm{en}\left(\frac{d+W}{\tau_{\mathrm{whloe}}}\right)$$

From Equations 2 and 3,

(4) $$-\mathrm{en}\left(\frac{d}{\tau_{\mathrm{SiNW}}}+\frac{W}{\tau_{\mathrm{Bulk}}}\right)=-\mathrm{en}\left(\frac{d+W}{\tau_{\mathrm{whole}}}\right)$$

The *τ*_SiNW_ was calculated by

(5) $$\tau_{\mathrm{SiNW}}=\frac{d}{\left(\frac{d+W}{\tau_{\mathrm{whole}}}-\frac{W}{\tau_{\mathrm{Bulk}}}\right)}$$

Figure 7 shows the lifetime of the SiNW arrays which was calculated from the Equation 5 as a function of the lifetime in the whole region when *d*, *W*, and *τ*_Bulk_ are 10 μm, 190 μm, and 1 ms, respectively. For confirmation of validation of this calculation, the *τ*_SiNW_ obtained by Equation 5 was compared to the simulation results of PC1D in Figure 7. We confirmed that the *τ*_SiNW_ using PC1D is in good agreement with the calculation based on Equation 5, and it was revealed that the *τ*_SiNW_ can be extracted by a simple equation such as Equation 5.

Finally, to estimate the optimal length of a SiNW for effective carrier collection, effective diffusion length of minority carriers was calculated from the obtained minority carrier lifetime. Most of the generated minority carriers have to move to an external circuit by diffusion because the depletion region of silicon solar cells is generally several hundred nanometers [37]. For simplification, SiNW arrays were regarded as a homogeneous film, and the measured carrier lifetime was assumed as the bulk lifetime of the homogeneous film. Effective diffusion length (*L*_*e*_) can be represented by

(6) $$L_{e}=\sqrt{\mathrm{D\tau}}$$

where *D* is the diffusion coefficient and *τ* is the bulk lifetime. From the Einstein relation, *D* is given by

(7) $$D=\frac{\mathrm{kT}}{q}\mu$$

where *k* is the Boltzmann constant, *T* is the absolute temperature, and *q* is the elementary charge. *μ* is the electron mobility of SiNW. The mobility of a SiNW depends on the length, diameter, and fabrication method. Therefore, we use an electron mobility of 51 cm^2^/(V s) because the SiNW array was fabricated by metal-assisted chemical etching in [25]. When Equation 6 is substituted in Equation 7, this yields the following expression for *L*_*e*_:

(8) $$L_{e}=\sqrt{\frac{\mathrm{kT}}{q}\mu \tau }$$

**Figure 8:**
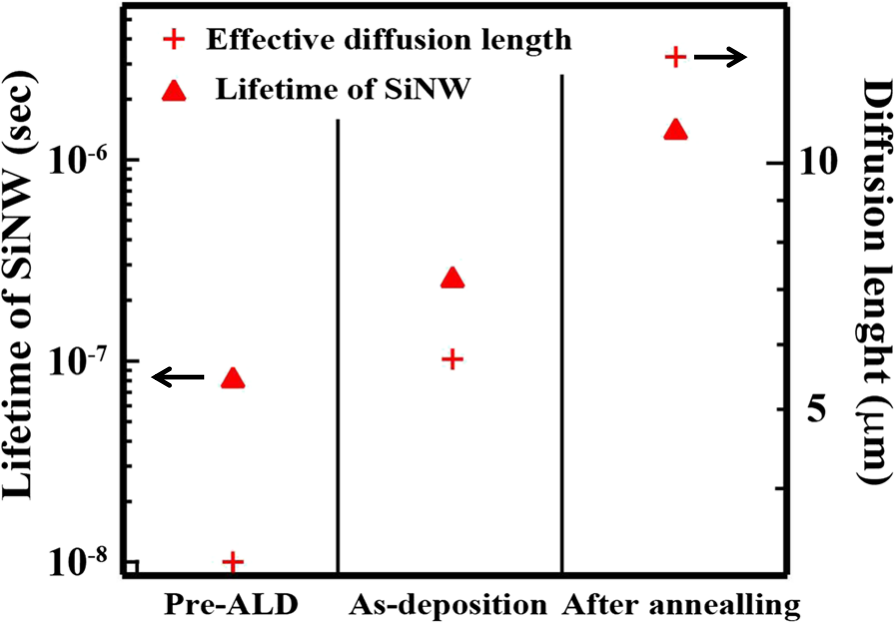
Lifetime and diffusion length in SiNW pre-ALD, as-deposited, and post-annealing.

Each value was substituted in Equation 8, and effective diffusion length was estimated at 3.25 μm without any passivation films (Figure 8), suggesting that minority carriers around the bottom of the SiNW arrays rapidly recombine, and that is why a very low carrier lifetime of 1.6 μs was obtained. In the case of Al_2_O_3_ deposited onto SiNW arrays, the diffusion length was estimated to be 5.76 μm, suggesting that passivation effect was not enough to collect minority carriers since there are defects still remaining. After annealing, the effective diffusion length improved to about 13.5 μm. In a heterojunction structure, a depletion region was formed between p-type amorphous Si layer and n-type SiNW. Photogenerated carriers in a SiNW diffuse into the electric region as diffusion current, reach the depletion region, and are collected as photocurrent. If the effective diffusion length is longer than the SiNW length, photogenerated carriers at the bottom region can be also collected as photocurrent. Since 13.5 μm is longer than the length, it is expected that most of the photogenerated carriers can be collected. Therefore, Al_2_O_3_ deposited by ALD is a promising passivation material for a structure with high aspect ratio such as p-type SiNW arrays. Moreover, it is effective to use a fixed charge in the passivation of SiNW arrays with dangling bonds.

## Conclusions

We successfully prepared SiNW arrays embedded in Al_2_O_3_ by using the MACES technique and the subsequent ALD deposition. HAADF-STEM clearly indicates that the SiNW was completely covered with Al_2_O_3_. This ALD-Al_2_O_3_ passivation film reduced surface recombination velocity at the surface of SiNW. The as-deposited Al_2_O_3_ increased minority carrier lifetime in the sample from 1.6 to 5 μs. Moreover, the lifetime improved up to 27 μs after annealing. These results indicate that ALD-Al_2_O_3_ is beneficial for the passivation of SiNW surfaces. In addition, we analyzed lifetime data in details to estimate minority carrier diffusion length of the SiNW region. According to the data analysis, we finally derived a simple analytical equation to extract the lifetime of the SiNW region from measured effective lifetime of the samples. Using the equation, it was found that the effective diffusion length of minority carriers in the SiNW array increased from 3.25 to 13.5 μm by depositing Al_2_O_3_ and post-annealing at 400°C. This improvement of the diffusion length is very important for application to solar cells. The larger diffusion length leads to better carrier collection in solar cells, and improvement of short-circuit current can be expected.

## Competing interests

The authors declare that they have no competing interests.

## Authors’ contributions

SK, YK, YW, and SM carried out the experiment and calculations. AY supervised the work and finalized the manuscript. YO, YN, and MH gave the final approval of the version of the manuscript to be published. All authors read and approved the final manuscript.
